# Prevalence of antibodies against seasonal influenza A and B viruses during the 2009-2010 and 2010-2011 influenza seasons in residents of Pittsburgh, PA, USA.

**DOI:** 10.1371/currents.RRN1265

**Published:** 2011-10-25

**Authors:** Ted M. Ross, Lu Hairong, Bing Shao Chia, Elaise Hill, Heather Weirback, Shanta Zimmer

**Affiliations:** ^*^University of Pittsburgh and ^¶^University of Pittsburgh, Center for Vaccine Research

## Abstract

Seroprevalence of antibodies against influenza viruses from 1000 people between the ages of 0 to 90 years of age (100 samples for each decade of life) in the Pittsburgh, PA, USA was measured. One year removed from the outbreak of novel H1N1 influenza into the human population in the Northern Hemisphere and following the emergence of a new H3N2 influenza isolate, sera was collected to determine the hemagglutination-inhibition antibodies against influenza A/H1N1, A/H3N2, and B viruses representative of viruses in the vaccine used for the 2010-2011 influenza season. The seroprevalence of antibodies to influenza virus, A/California/7/2009 (H1N1), increased from the previously reported November 2009 samples and the samples collected at the end of the 2010 influenza season (June 2010) during the 2010-2011 season in all age groups, but people the under the age of 20 had the highest rise in the number of positive samples. The number of individuals positive for H1N1 stayed the same through the entire influenza season. In contrast, there were little to no positive serum samples against the H3N2 virus, A/Perth/16/2009, from samples collected during the 2009-2010 influenza season, however, titers against these viruses rose significantly during the early months of the 2010-2011 season with the highest number of positive samples detected in the very young and very old populations. However, these titers waned by May, 2011 in those over the age of 40. There was a rise in adults to the B/Brisbane/60/2008 influenza virus in adults in samples collected in October, 2010, but these titers quickly declined. The highest titers to B influenza were detected in people between the ages of 10-30 years of age. These findings may have implications for the development of vaccination strategies aiming at the protection against seasonal and/or pandemic influenza virus infection and pre-pandemic preparedness activities.

## **Introduction**

Influenza A virus (IAV) and B are the cause annual outbreaks resulting in significant illness and loss of economic productivity.  The viral antigens hemagglutinin (HA) and neuraminidase (NA) are the immune protective targets of the virus and changes (antigenic shift and antigenic drift) in these HA and NA molecules results in evasion of the immune system.  Although influenza infection is generally associated with seasonal epidemics (during the winter months), the virus can be detected in a large city throughout the year and is identified year round in various parts of the world [1].  IAV transmits more efficiently in the cold and dry atmospheric conditions that exist in the winter months which may explain the pattern of seasonal epidemics [2].  Each influenza season, 200,000 hospitalizations and 36,000 excess deaths are attributed to influenza outbreaks annually [3].  Even though children and the elderly are equally infected with influenza each season, the vast majority of deaths occur in the elderly population.  However, children are more likely to spread to other children and therefore, may have the highest incidence of infection following the introduction of a newly emerged influenza virus [4].  Children have higher viral loads [5] [6] and longer periods of shedding, allowing efficient transmission and spread of influenza.

                Vaccination against influenza provides a potent and cost-effective counter-measure to the threat of both seasonal and pandemic outbreaks. Licensed seasonal influenza vaccines are only partially protective, particularly in the elderly and young children.  In April 2009, a new pandemic strain of H1N1 influenza emerged and spread rapidly throughout the globe [1]. A second wave of pandemic H1N1 swept through the United States. Our research group previously characterized the seroprevalence of people living in Allegheny County (Pittsburgh), Pennsylvania as the epidemic peaked in late-October in a largely unvaccinated community [7].  Seroprevalences against pandemic 2009 H1N1 influenza varied by age group, with children age 10–19 years having the highest seroprevalence (45%), and persons age 70–79 years having the lowest (5%). The baseline seroprevalence among control samples from 18–24 year-olds was 6%. Measurement of the seroprevalence of influenza immunity provides valuable information about the likelihood of possible influenza spread, vaccine effectiveness and may be useful in decision-making about immunization strategies.  

                In order to enhance vaccine prepardeness, we continued these seroprevalence studies throughout the 2010-2011 influenza season.   For this season, a new strain of H3N2 influenza, A/Perth/16/2009, was used in the vaccine to better match circulating viruses.  These studies gave us the opportunity to follow the seroprevalence both for the newly emerged H1N1 for another season, but also the development of antibodies in the human population to a new H3N2 vaccine strain.

## **Methods**

### Sample cohorts and collections

The samples analyzed were excess serum samples collected anonymously from extra laboratory specimens from the University of Pittsburgh Medical Center's Presbyterian Hospital and the Children's Hospital of Pittsburgh at 5 different time points in November 2009, June 2010, October 2010, February 2011, and May 2011.  University of Pittsburgh IRB approval [(exempt) #PRO09110164] was obtained before collection.  The November 2009 collections occurred ~2–4 weeks after the peak of the novel H1N1 fall wave of infections and the June 2010 collections occurred two months past the end of the traditional influenza season.  Three additional collections time points were performed for the 2010-2011 influenza season at the beginning (October 2010), at the peak (February 2011), and at the end (May 2011).   Serum samples were collected anonymously from ~1000 persons (100 samples for each decade) at each time collection that ranged in age from 1 month to 90 years of age using the honest broker system at the University of Pittsburgh Laboratories and given to investigators organized by decade of birth without other identifying information. Each serum sample was classified by decade of birth of the donor and tested in hemagglutination-inhibition assay (HAI) against pandemic H1N1 (A/California/7/2009), H3N2 (A/Perth/16/2009), and B/Brisbane/60/2008 (Victoria lineage).  Reference sera from individuals vaccinated with either inactivated trivalent seasonal Fluzone vaccine or pandemic H1N1 FluMist (GSK) vaccines were used as positive controls. 

### Hemagglutination-inhibition (HAI) assays

Hemagglutination inhibition (HAI) assays were conducted as previously described [8] [9], . To inactivate non-specific inhibitors, aliquots of each serum sample were separately treated with receptor destroying enzyme (RDE) prior to being tested with a final serum dilution of 1:10 (starting dilution for the assays). Samples were serially diluted 2-fold into V-bottom 96-well microtiter plates. An equal volume of virus, adjusted to approximately 8HAunits/50 microliter was added to each well. The plates were covered and incubated at room temperature for 30 min followed by the addition of freshly prepared 1% turkey erythrocytes (RBCs) (Lampire Biologicals, Pipersville, PA, USA) in phosphate buffered saline (PBS). The plates were mixed by agitation, covered, and allowed to set for 30 min at 25°C. The HAI titer was determined by the reciprocal of the last dilution which contained non-agglutinated RBCs. Positive and negative serum controls were included on each plate. Samples with HAI titers ≥1:40 were considered seropositive.

### Sample Size Calculations

Sample size calculations were performed based on an estimated seroprevalence of 30% indicated that 89 samples would be required per decade to detect seroprevalence +/−10% within a 95% confidence interval.****

### Statistical analysis

Geometric mean HAI titers and standard error were calculated for each group. Sensitivity analysis was conducted around HAI titer cut offs of 1:40, 1:80 and 1:160 (data not shown) before the decision was made to use the conventional 1:40 as the cut off value for seropositivity.  Cochran-Armitage test for trend was calculated across age groups.

## **Results**

### **Influenza cases**

                In late April 2009, at end of the usual influenza season in the Northern Hemisphere, the first two cases of swine influenza H1N1 were identified in the United States.   The Centers for Disease Control and Prevention confirmed that these cases were both caused by a genetically similar swine virus that had not been previously recognized in the United States [10].  The number of cases declined during the summer months, but, there was a second peak of influenza cases in November 2009 that declined by January, 2010.  This unusual influenza season was preceded (2008-2009) and then followed (2010-2011) by typical patterns of influenza cases detected during the influenza season (Fig. 1).  Interestingly, the new H3N2 viruses that were A/Perth/16/2009-like were detected earlier in the season than H1N1 or B influenza viruses (Fig. 2).  A rise in the number of influenza reported cases in the United States in December, 2010 were of the H3N2 subtype, whereas H1N1 and influenza B cases began to rise a month later.  For all three subtypes, the number cases peaked during the 9^th^ week of 2011.  Influenza B represented ~20% of all the reported influenza cases.  By the 16^th^ week (end of April, 2011), the number of cases had declined to baseline.

**Figure:**
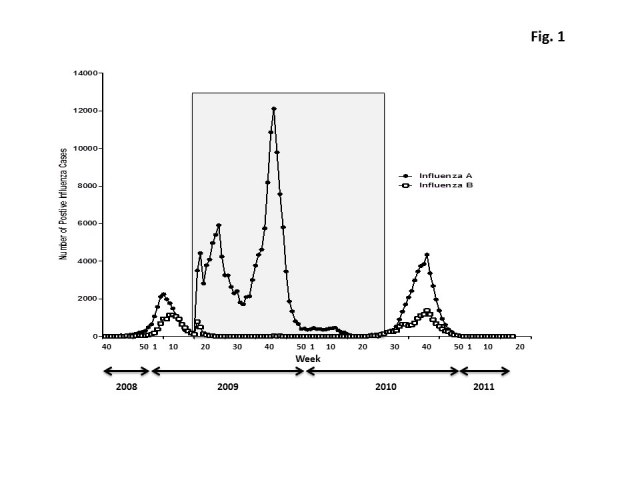
Figure 1. Distribution of the influenza A and B cases in the US population from week 40 in 2008 to week 17 in 2011.

**Figure:**
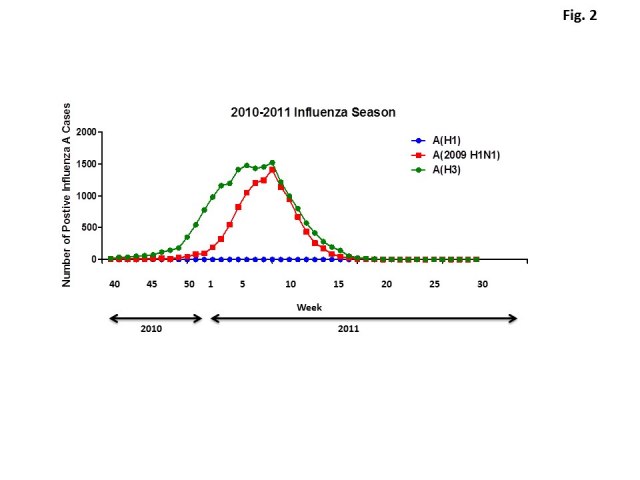
Figure 2. Number of influenza A cases by subtype (H1N1, 2009(H1N1), and H3N2) from week 40 in 2009 to week 29 in 2011.

### **Seroprevalance of study population:  1920-2011**

Serum samples were collected from individuals in Allegheny County, Pennsylvania, USA at 5 different time points from November 2009 to May 2011.  The November 2009 collections occurred ~2–4 weeks after the peak of the novel H1N1 fall wave of infections and the June 2010 collections occurred two months past the end of the traditional influenza season.  Three additional collections time points were performed for the 2010-2011 influenza season at the beginning (October 2010), at the peak (February 2011), and at the end (May 2011).   Serum samples were collected anonymously from ~1000 persons at each time collection that ranged in age from 1 month to 90 years of age. As previously reported, the percentage of persons with serum that tested positive for pandemic H1N1 influenza (A/California/7/2009) in November 2009 was highest among children in the 10–19 year old age group (46%) and the 0–9 year-old age group (29%). These results were similar in samples collected in June2010, except the number of people positive for novel H1N1 doubled in people in the 1940s, 1970s, and 1980s compared to the November 2009 time period (Fig. 3A).  In addition, the number of children under the age of 10 testing positive had dropped from 28% in November 2009 to 13% in June 2010.  The percentage of people positive for novel H1N1 rose to 35-40% in people born between 1920 and 1979 that was maintained through the February and May 2011 collections.  There was a sharp rise in percent of positive serum samples in people born after 1980 with >50% of these samples testing positive in HAI assay to novel H1N1.  

**Figure:**
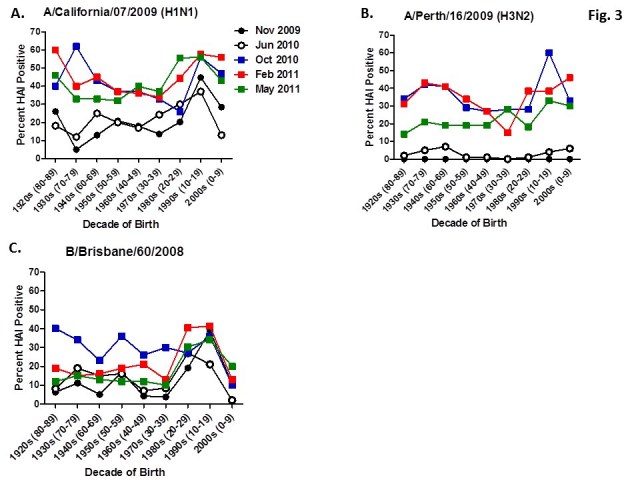
Figure 3. Percent seropositive (HAI>1:40) by decade of birth for (A) A/California/7/2009, (B) A/Perth/16/2009, and (C) B/Brisbane/60/2008.

For the 2010-2011 influenza season, the Advisory Committee on Immunization Practices (ACIP) recommended a change in the strain of H3N2 used in the trivalent influenza vaccine, A/Perth/16/2009 [11].

## **Discussion**

Previously, our research group examined the seroprevalence of 2009(A)H1N1 influenza antibodies shortly after the fall wave (considered the second pandemic wave) in the Allegheny County (Pittsburgh, PA) United States (Zimmer).  That study was a part of a number of seroprevalence studies performed in different locations around the world published in 2010 [8] [9] [12] [13] [14] [15] [16] [17] [18] [19].  Overall, these studies showed that children had the highest percentage of people with antibodies to this newly emerged H1N1 virus, but that older individuals, particularly those over 60 years of age, may have cross-reactive antibodies to this novel H1N1 influenza [12] [17] [20] [21] [22] [23] [24].

During the 2010-2011 influenza season, we expanded these studies to include not only the prevalence of antibodies to the novel H1N1, but also the H3N2 and B influenza virus.  Samples collected after the 2009-2010 influenza season (June2010), had similar numbers of HAI positive samples against the novel H1N1 by age as detected during the second wave (November 2009).  Children, under the age of 19 still had the highest percentages of individuals infected against all three strains tested.  The percentage of positive HAI samples doubled in almost all age groups and was maintained throughout the 2010-2011 season.  These results are supported by a study of individuals in Scotland where the seroprevalance shifted upwards against novel H1N1 in relation to 2010 [8] [21].

A new H3N2 strain, A/Perth/16/2009, was selected for the trivalent influenza vaccine in 2010-2011.  Almost no individuals, regardless of age, had pre-existing antibodies to this new H3N2 strain (Fig 3B).  By October 2010, there was a sharp rise in the number of positive serum samples with 30-45% of the serum samples were positive.  Most likely, these positive H3N2 samples were due to vaccination, not infection, since cases of influenza Perth/09-like viruses were not detected December 2010.  The number of positive HAI titers to H3N2 was maintained in sera collected in February2011, but declined particularly in older people by May 2011, which may indicate waning immune responses elicited by the vaccine. The number of B influenza positive samples increased by October 2010, which may also be a result of vaccination.  However, by February 2011, the percentage of positive samples fell back to mid-2010 levels for most groups, except for people born in the 1980s and 1990s which may indicate antibodies elicited in response to infection.  For all three influenza strains, there are intense social mixing patterns in school age children that drive transmission of respiratory viruses, and make children highly likely to be infected with influenza, particularly a newly emerged influenza strain .

These serum samples were collected anonymously from discarded laboratory specimens from the University of Pittsburgh Medical Center's Presbyterian Hospital and the Children's Hospital of Pittsburgh.  Except for decade of birth, no other data was collected from any patient, such as potential high risk group characteristics or vaccination status.  Even though we were unable to separate vaccine induced responses from virus induced responses, it appears that most of the H3N2 and B influenza responses in people over 40 were induced by vaccination, since the number of positive people declined by the end of influenza season.  The titers were, for the most part, maintained in teenagers and 20 year olds, which is the target group most likely infected.  Increased seropositivity in the ≥40 year olds between 2009-2010 and 2010-2011 seasons is strongly correlated with vaccination and may suggest that compared to younger individuals, that infection rates were lower in the older age group.

This study continues the antibody seroprevalence analysis following the 2009 pandemic and is one of the first assessments during 2010-2011 influenza season in the northern hemisphere.  Serosurveillance is one of the best ways to estimate influenza infections at the population level.  As seen during the 2009 H1N1 pandemic, some people that seroconverted to this virus were most likely asymptomatic [12] [13].  Serology information allows for estimates of at risk populations and assists with future pre-pandemic planning initiatives.  Studies, such as the one presented here, help prepare for the next pandemic by providing data on which populations should be vaccinated, such as children, people with underlying conditions, military personnel, and health care workers.  The data shows how many people have neutralizing or HAI antibodies against circulating strains and how those antibodies are being maintained in the population.  It is not known when or what subtype may cause the next pandemic outbreak, but ongoing seroprevalence studies can capture a population’s pre-existing immunity and establish baselines for detecting any changes in the rate both during pandemics and during interpandemic periods.

**Figure:**
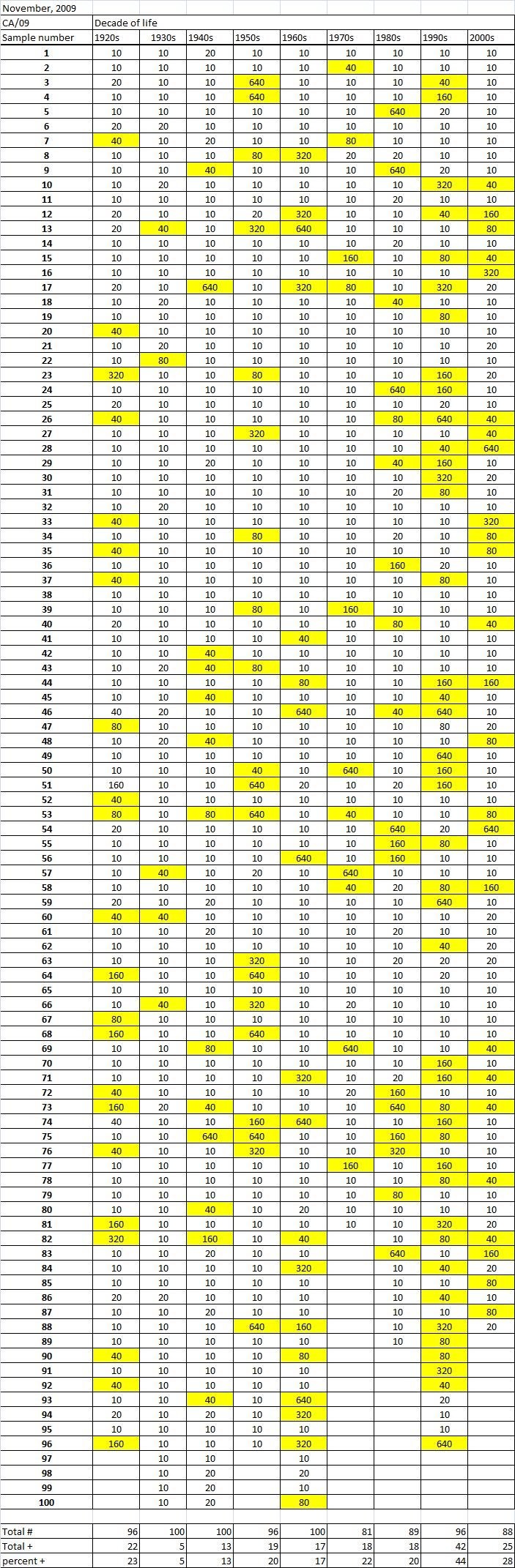
Table 1A

**Figure:**
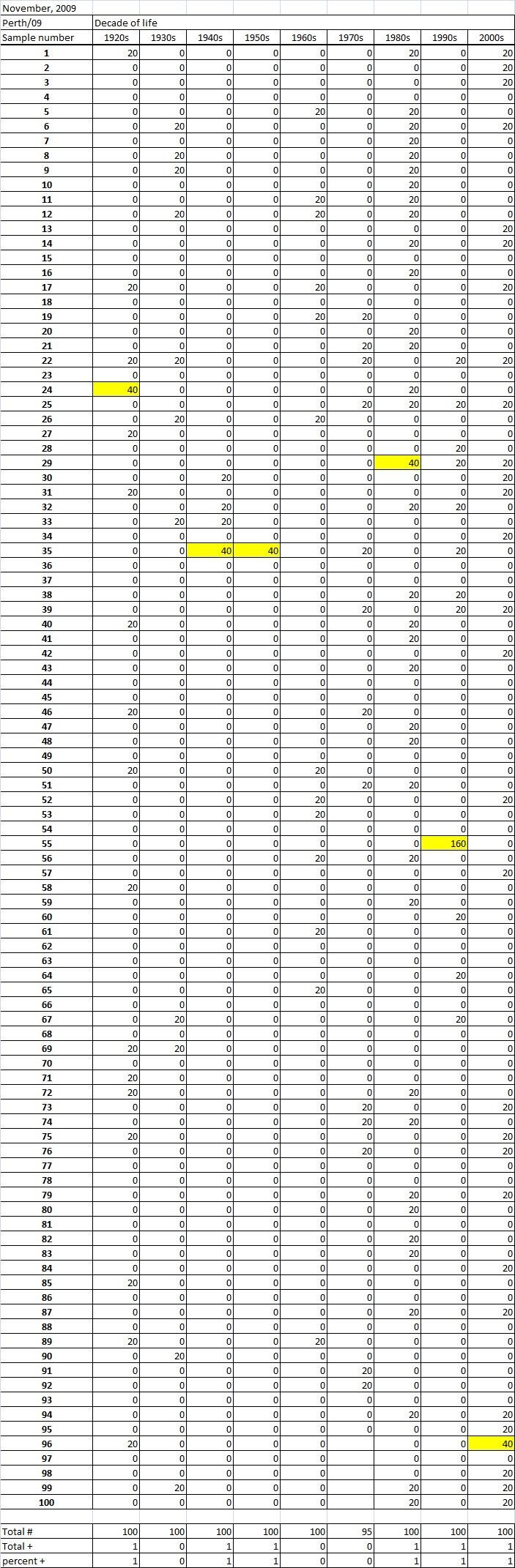
Table 1B

**Figure:**
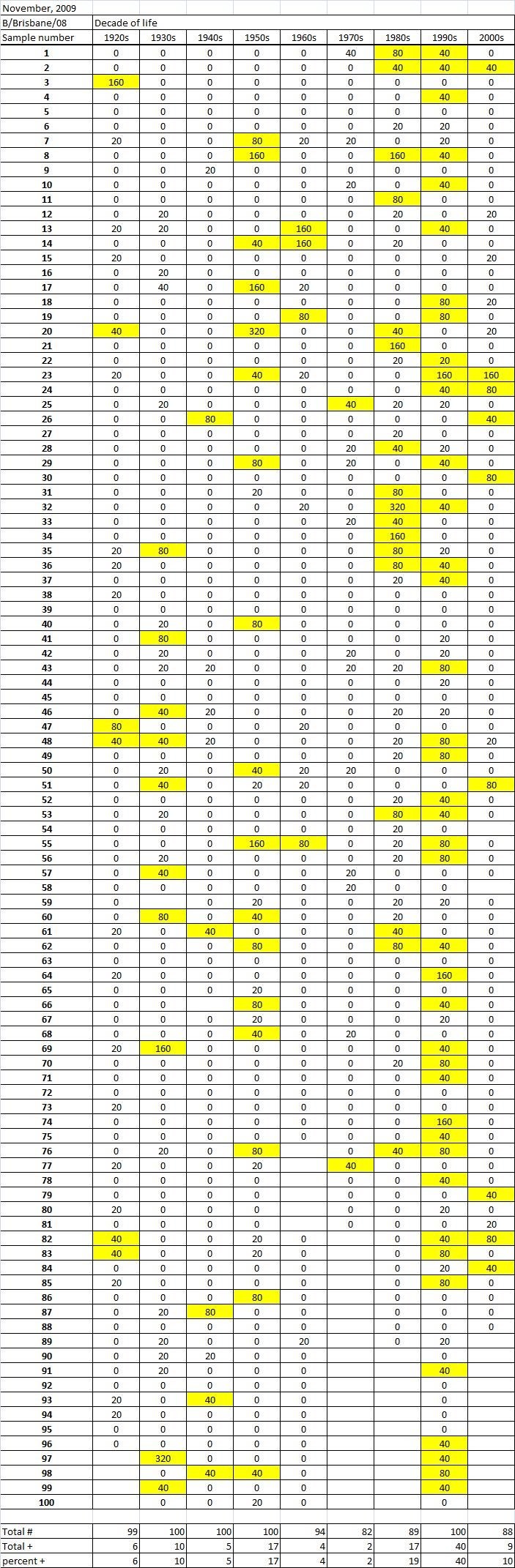
Table 1C. Table 1.  Hemaggltunination-inhibition titers from serum samples collected in November, 2009 against A/California/7/2009 (H1N1), A/Perth/16/2009 (H3N2), B/Brisbane/60/2008. 

**Figure:**
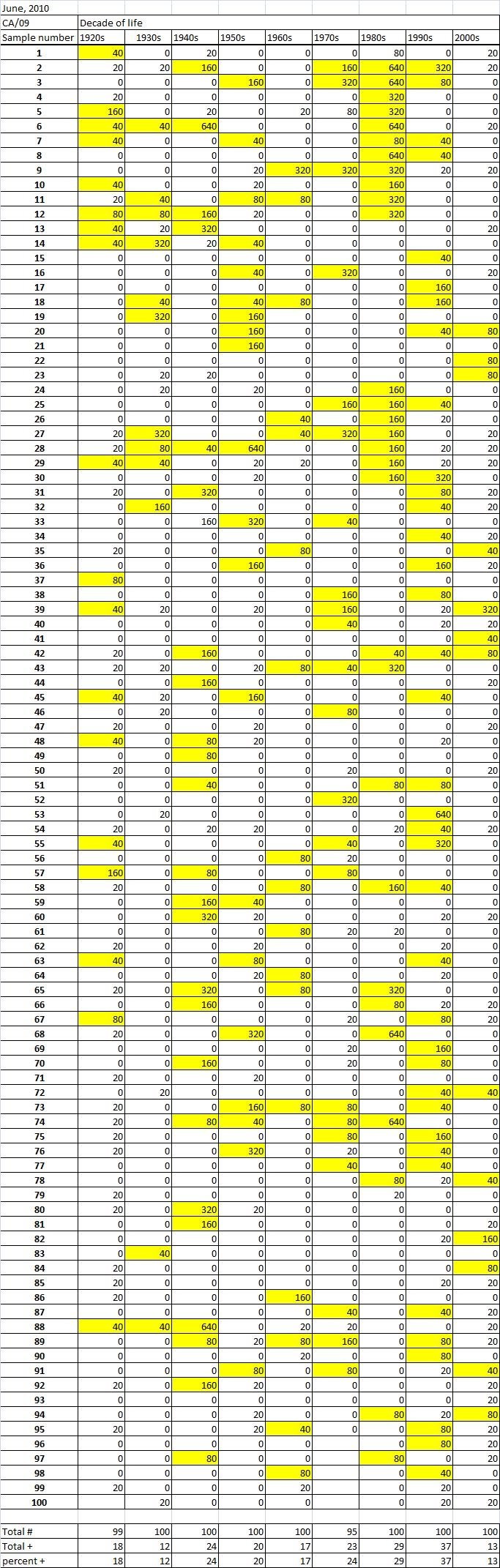
Table 2A 

**Figure:**
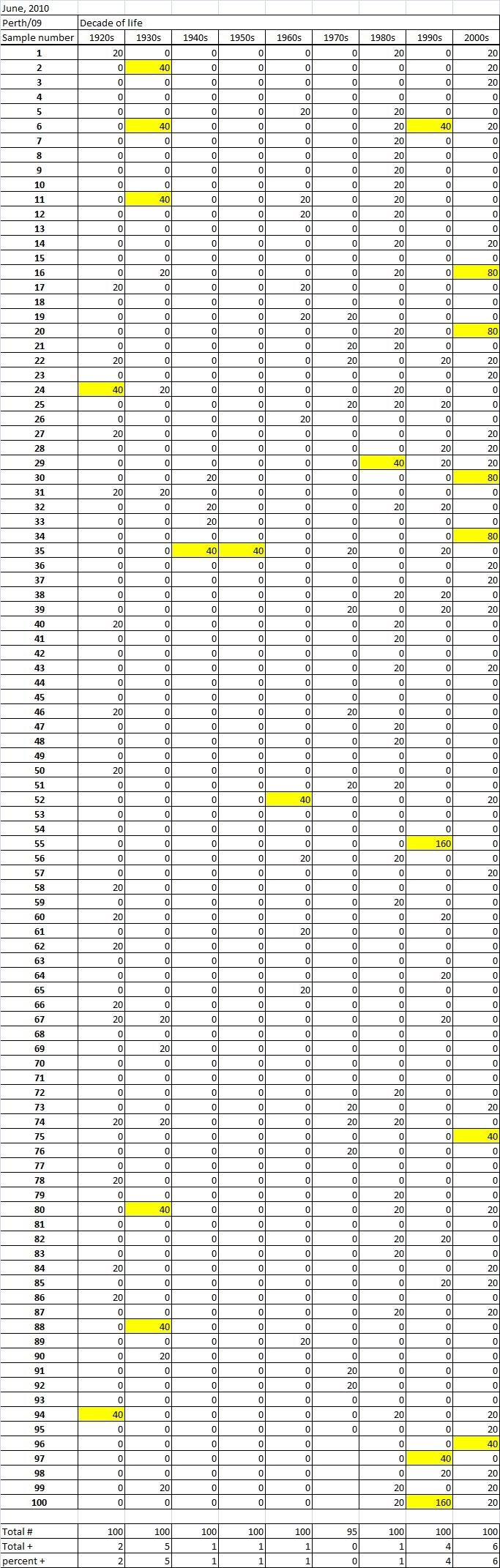
Table 2B 

**Figure:**
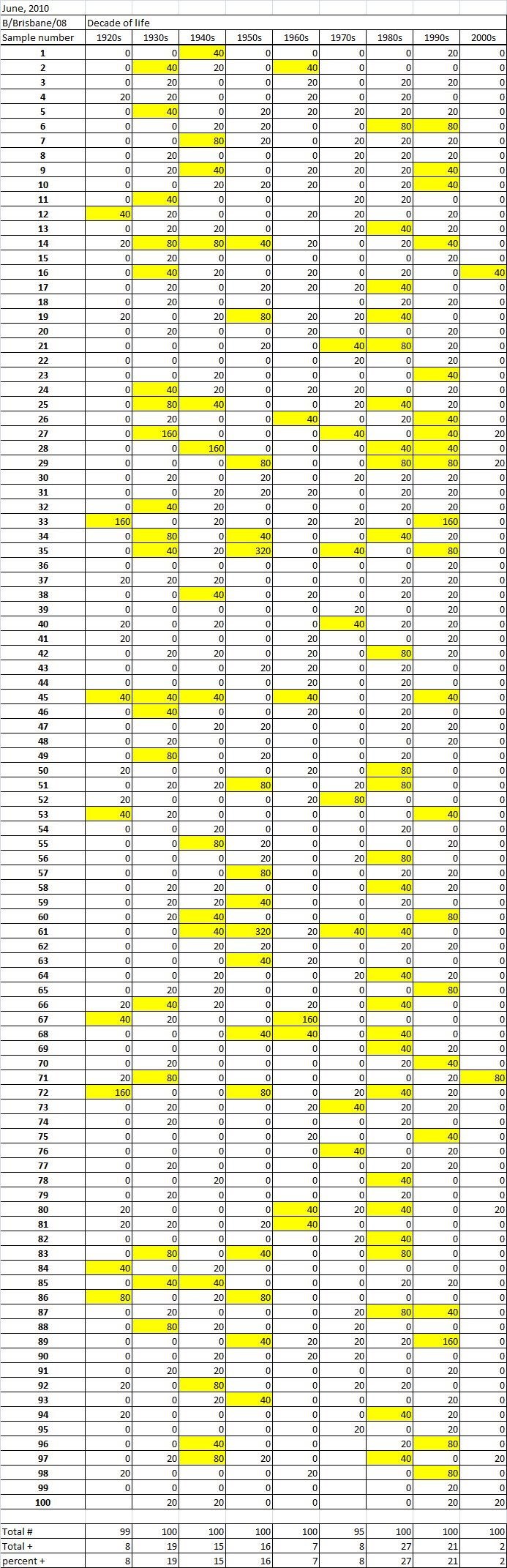
Table 2C. Table 2.  Hemaggltunination-inhibition titers from serum samples collected in June, 2010 against A/California/7/2009 (H1N1), A/Perth/16/2009 (H3N2), B/Brisbane/60/2008

**Figure:**
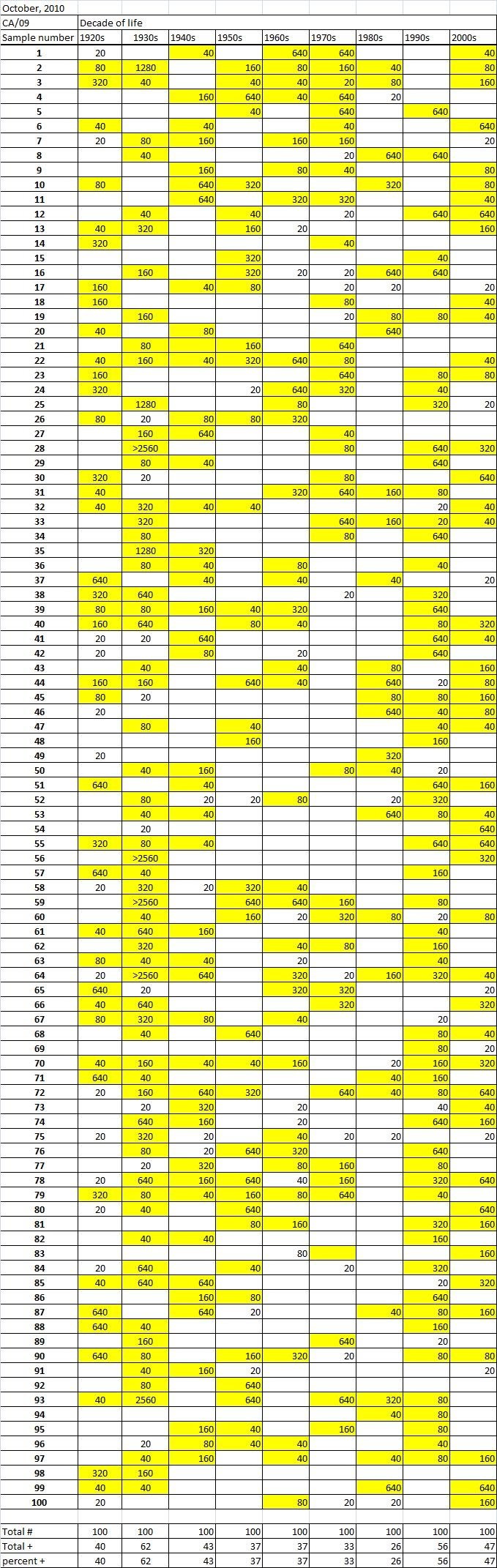
Table 3A

**Figure:**
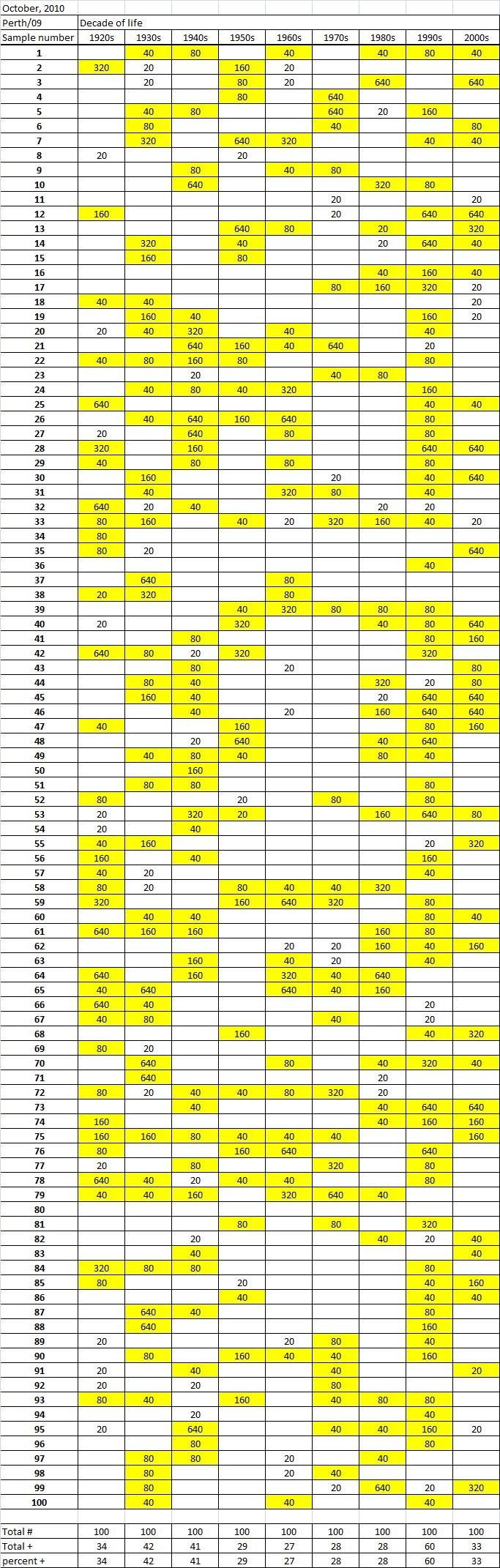
Table 3B 

**Figure:**
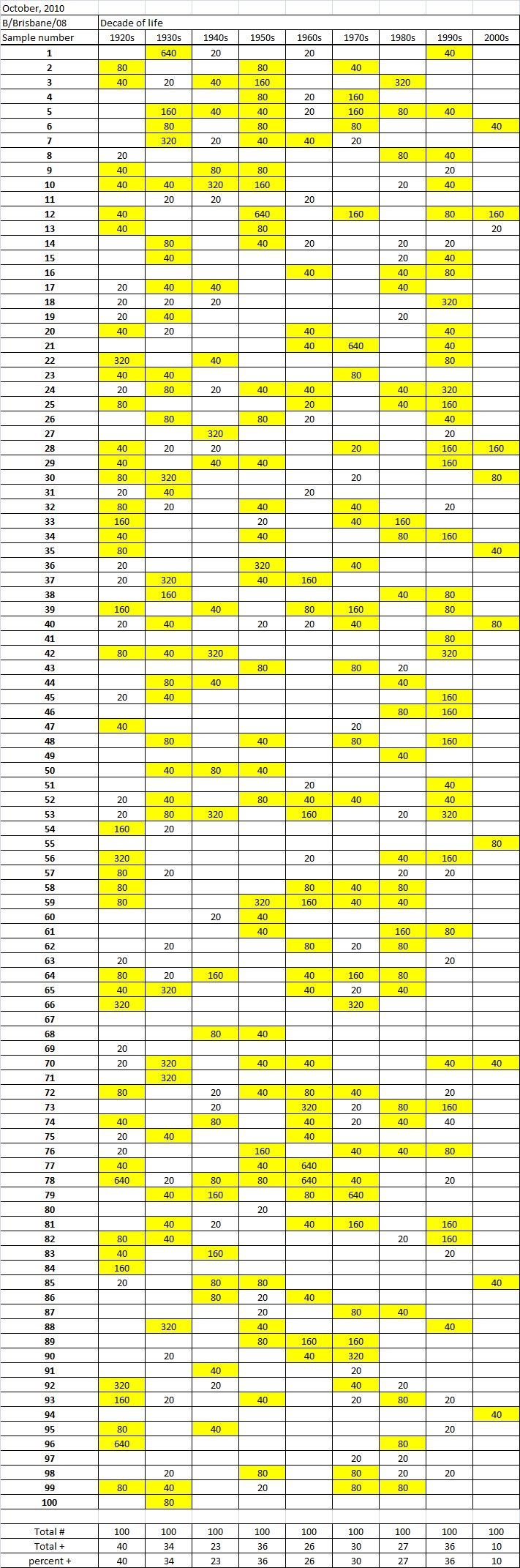
Table 3C. Table 3.  Hemaggltunination-inhibition titers from serum samples collected in October, 2010 against A/California/7/2009 (H1N1), A/Perth/16/2009 (H3N2), B/Brisbane/60/2008

**Figure:**
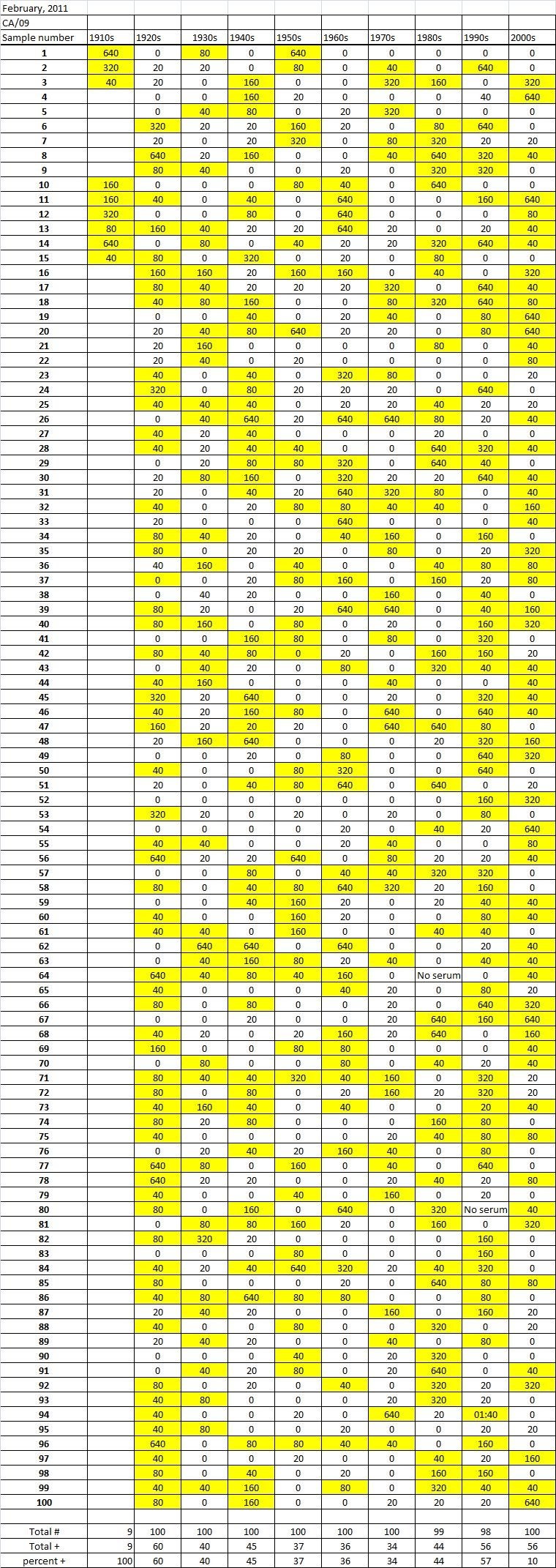
Table 4A

**Figure:**
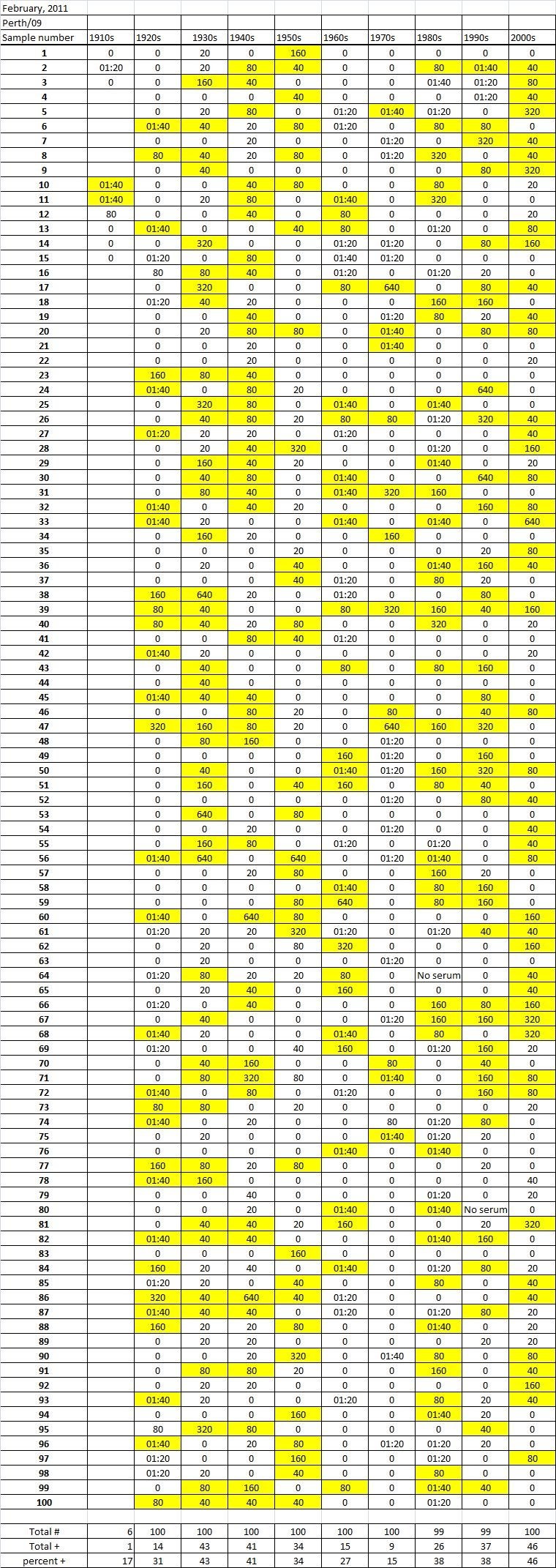
Table 4B

**Figure:**
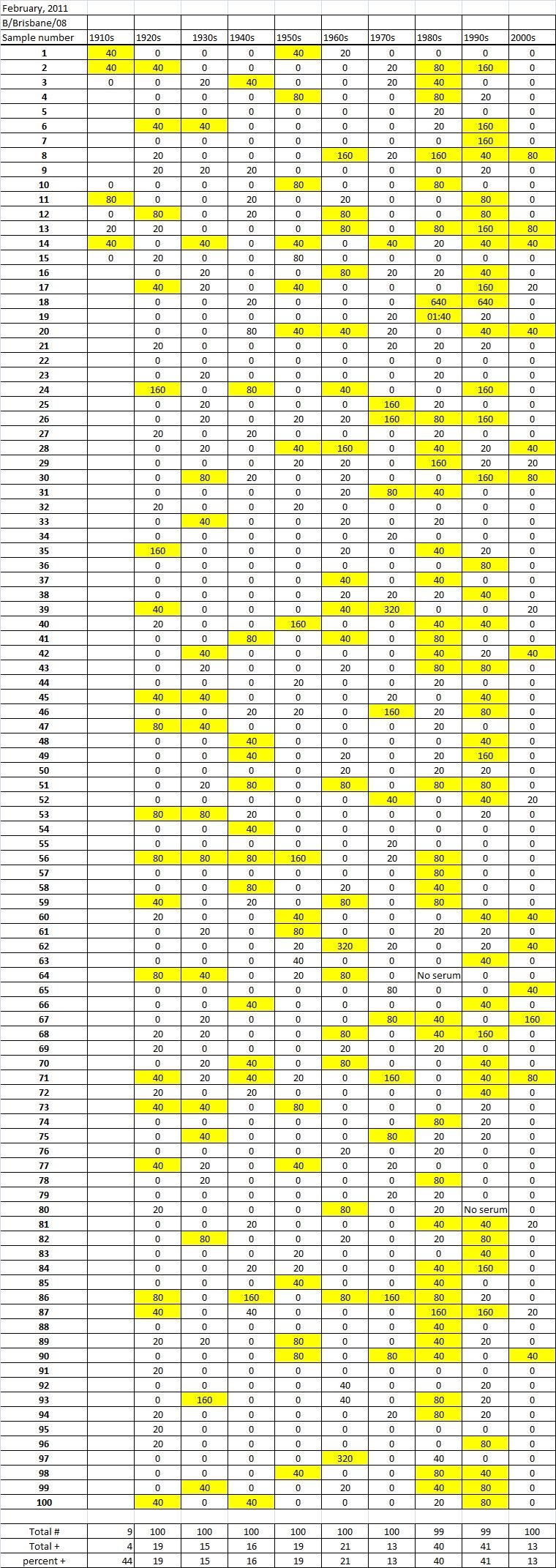
Table 4C. Table 4.   Hemaggltunination-inhibition titers from serum samples collected in February, 2011 against A/California/7/2009 (H1N1), A/Perth/16/2009 (H3N2), B/Brisbane/60/2008

**Figure:**
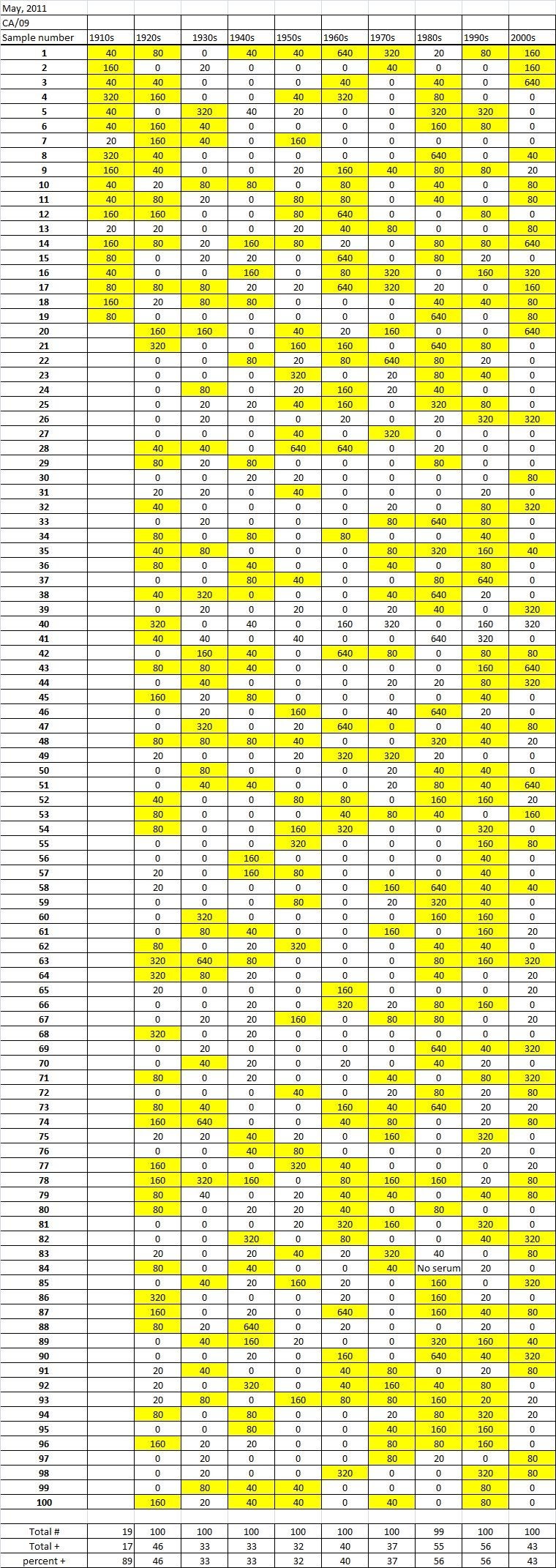
Table 5A

**Figure:**
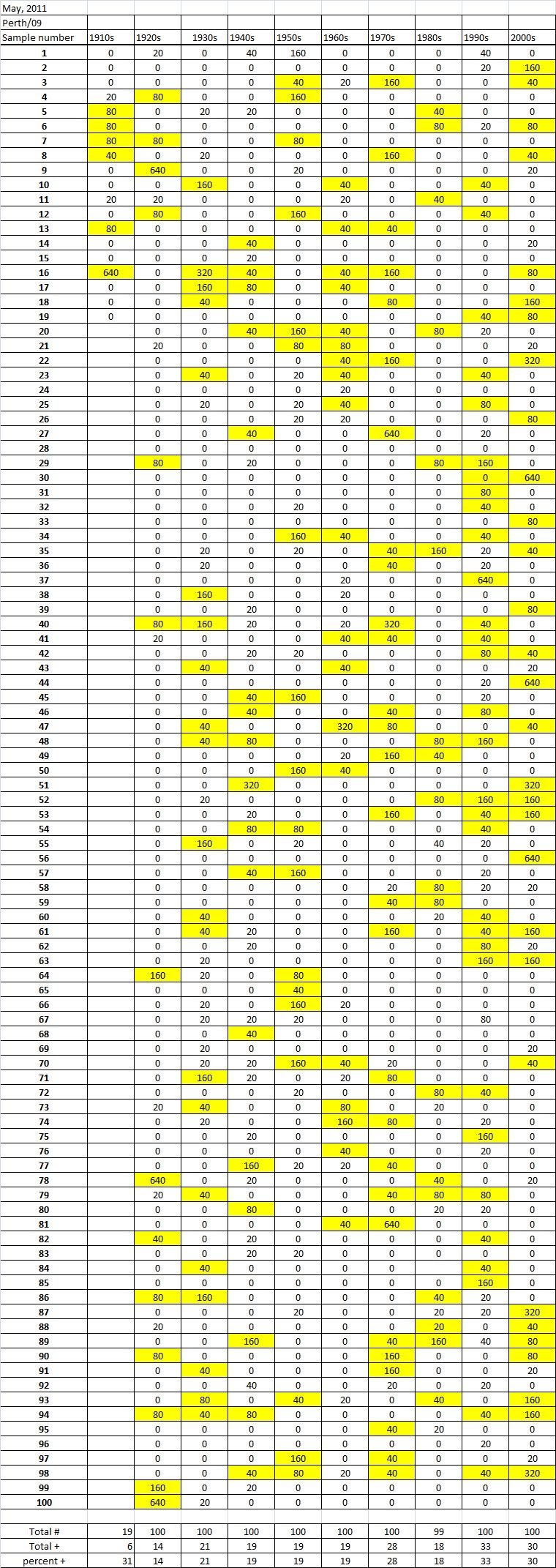
Table 5B

**Figure:**
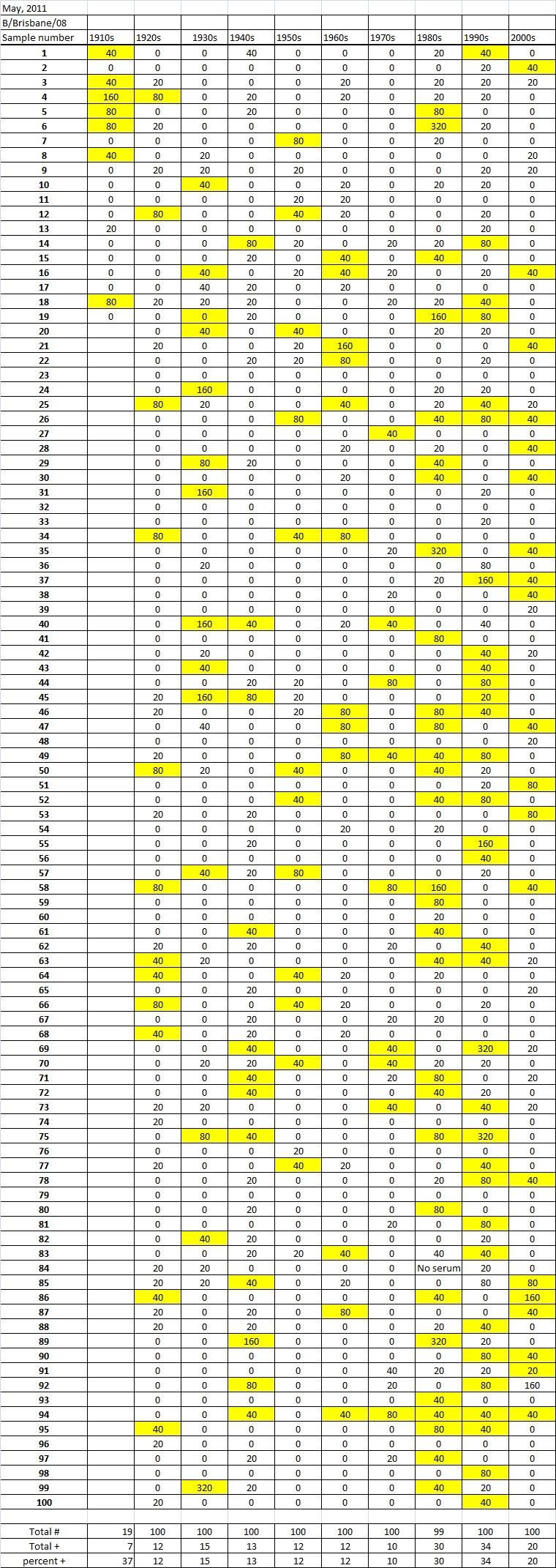
Table 5C. Table 5.  Hemaggltunination-inhibition titers from serum samples collected in May, 2011 against A/California/7/2009 (H1N1), A/Perth/16/2009 (H3N2), B/Brisbane/60/200

##  Acknowledgements

The authors would like to thank the staff of the laboratories of the University of Pittsburgh Medical Center for their assistance with sample acquisition, and Steve Wisniewski for initial sample size calculations. 

## **Funding information**

The authors thank the University of Pittsburgh for funding support.

## **Competing interests**

The authors have declared that no competing interests exist. 

## **Author Contributions**

Conceived and designed the experiments: SMZ, TMR. Performed the experiments: HRL, EH, BSC, HRW. Analyzed the data: SMZ, HRL, TMR. Contributed reagents/materials/analysis tools: EH, BSC, HRW, TMR. Wrote the paper: SMZ TMR.
